# Impact of perinatal hypertonic NaCl access on adult offspring’s sodium intake and angiotensin and vasopressin systems under hypertension model

**DOI:** 10.1038/s41598-025-93238-4

**Published:** 2025-03-15

**Authors:** Cintia Y Porcari, Cristina A Lencina, José L Amigone, José Antunes-Rodrigues, Ximena E Caeiro, Andrea Godino

**Affiliations:** 1 Instituto de Investigación Médica Mercedes y Martín Ferreyra, INIMEC-CONICET-Universidad Nacional de Córdoba, Friuli 2434, Córdoba, 5016 Argentina; 2 Facultad de Psicología, Universidad Nacional de Córdoba, Boulevard de la Reforma & Enf. Gordillo Gómez, Córdoba, 5000 Argentina; 3 Department of Physiology, School of Medicine of Ribeirao Preto, University of Sao Paulo, Av. Bandeirantes 3900, Ribeirão Preto, CEP:14049-900 Brazil; 4 Sección de Bioquímica Clínica, Hospital Privado, Córdoba, Argentina

**Keywords:** Perinatal programming, Blood pressure regulation, Hypertension, Angiotensin type 1 receptor, Voluntary sodium consumption, DOCA-salt, Developmental biology, Physiology

## Abstract

Perinatal natriophilia has programming effects on blood pressure control, inducing anatomical and molecular changes in the kidney and brain that impair blood pressure reestablishment after a pressor challenge, such as an osmotic stimulation. However, the imprinted effect of voluntary sodium consumption during this period on the development of hypertension is unclear. To evaluate this, we studied the effect of deoxycorticosterone acetate and high-salt diet (DOCA-salt) treatment on blood pressure and sodium intake responses, and gene expression in the kidney and brain in adult offspring exposed to voluntary hypertonic sodium consumption during the perinatal period (PM-NaCl group). Male PM-NaCl rats consumed more sodium than controls (PM-Ctrol group) during DOCA treatment. However, the hypertension induced did not differ between the PM-NaCl and PM-Ctrol groups. This behavioral change was accompanied by a higher angiotensin type 1 receptor (*Agtr1a*) gene expression at brain level in the subfornical organ and the hypothalamic paraventricular nucleus of PM-NaCl, areas key to the modulation of salt appetite and autonomic function. At renal level, programmed animals showed differing responses in gene expression induced by DOCA-salt treatment compared to the PM-Ctrol group, such as expression of *Agtr1a*, transient receptor potential vanilloid type 1 channel in the medulla and vasopressin 2 receptor in the renal cortex. The data indicates that the availability of a rich source of sodium during the perinatal period induces a long-term effect in DOCA-salt treated rats, modifying behavioral, brain and renal responses, suggesting that this early sodium exposure affects the vulnerability of the organisms to chronic non-communicable diseases mainly caused by changes in sodium intake and the regulatory mechanisms of the angiotensin and vasopressin systems.

## Introduction

Hypertension (HTN) is a prevalent condition in both developed and developing nations, significantly increasing the risk of cardiovascular events and mortality. Its etiology is complex, involving genetic, epigenetic, and environmental factors that contribute to chronic blood pressure (BP) elevation. Although over 120 loci linked to BP regulation have been identified, they account for only a small fraction of HTN inheritance^1^. Research indicates that HTN is often associated with early perinatal experiences, sympathetic nervous system overactivity, and renin-angiotensin system (RAS) imbalances^1,2^.

Recent studies have shown that a forced-high-sodium diet during the perinatal period leads to lasting increases in sodium consumption and blood pressure in adult offspring^3–5^. The mechanisms by which early life events influence the development of hypertension have not been fully elucidated, but it is likely that the number of glomeruli, and functional changes in post-glomerular segments of the nephron that participate in sodium handling, play an important role^6^.

Salt sensitivity hypertension involves an alteration in the blood pressure-sodium excretion relationship or “pressure natriuresis”, which shifts to the right, meaning that higher blood pressure is required to maintain normal salt excretion^7,8^. Several mechanisms, many affected epigenetically during the early stages of development, have been considered to promote salt sensitivity in adults, including a nephron/glomeruli deficit (reduced kidney mass) or changes related to the renin-angiotensin-aldosterone system [such as angiotensin II (ANGII), angiotensin type I and II receptors, AT1R and AT2R, respectively] and renal Na + transporters^9^. It has been demonstrated that kidney salt-inducible kinases (SIK)^10^ modulate transcriptional pathways in response to physiological signals, such as dietary sodium, feeding/fasting, and inflammation. The distal tubule segment is a key site of hormone-regulated Na + absorption, and it has been demonstrated that the epithelial sodium channel (ENaC) participates in the initiation and progression of salt-sensitive hypertension^11^. On the other hand, vasopressin (AVP) induces antidiuresis, by V2 vasopressin receptors (V2R) through regulation of the water channel, aquaporin-2 (AQP2), and is also involved in the activation of two structurally related Na-(K)-Cl co-transporters, promoting Na reabsorption in the distal nephron to concentrate urine^12^. In contrast, transient receptor potential vanilloid type 1 channel (TRPV1) activation, at renal level, increases the glomerular filtration rate, thus having a protective role on the blood pressure increase^13^. All these elements play an essential role in body water and electrolyte homeostasis, and their alterations have been associated with the salt-sensitive phenotype.

Deoxycorticosterone acetate combined with salt treatment (DOCA-salt model) is ideal for delineating the role of these main pathways that are critical in the pathogenesis of hypertension as it generates an imbalance of renal sodium, increasing the reabsorption of water and sodium, and leading to a state of hypervolemia^14,15^.

Similarly, our recent results demonstrated that animals programmed with ad libitum hypertonic sodium solution, based on the natriophilia proper to the perinatal period, had altered BP regulation, lower glomerulus number, lower renal expression of transient receptor potential cation channel (*Trpv1*), higher renal expression of angiotensin type 1a receptor (*Agtr1a*), and also lower expression of *de novo* synthesized AVP after osmotic challenge^16^. Therefore, we hypothesize that perinatal imprinting induced by voluntary hypertonic sodium consumption (natural natriophilic state) induces a salt-sensitive phenotype that alters the threshold or window of onset of hypertension induced by DOCA-salt protocol. Thus, our aim was to analyze the BP progression, and renal expression of the channels (TRPV1, ENaC) and receptors (AT1R, AT2R and V2R) involved in water/sodium excretion after DOCA treatment in early sodium-programmed and control animals. Finally, in the same groups, we analyzed at brain level the expression of AT1R and serotonin type 2 C receptor (5HT2CR), previously associated with the stimulation and inhibition of sodium consumption respectively^17–19^ induced by DOCA-salt protocol.

## Results

### Effects of sodium programming on body, brain, heart, and kidney size induced by DOCA-salt treatment

**Fig. 1:**
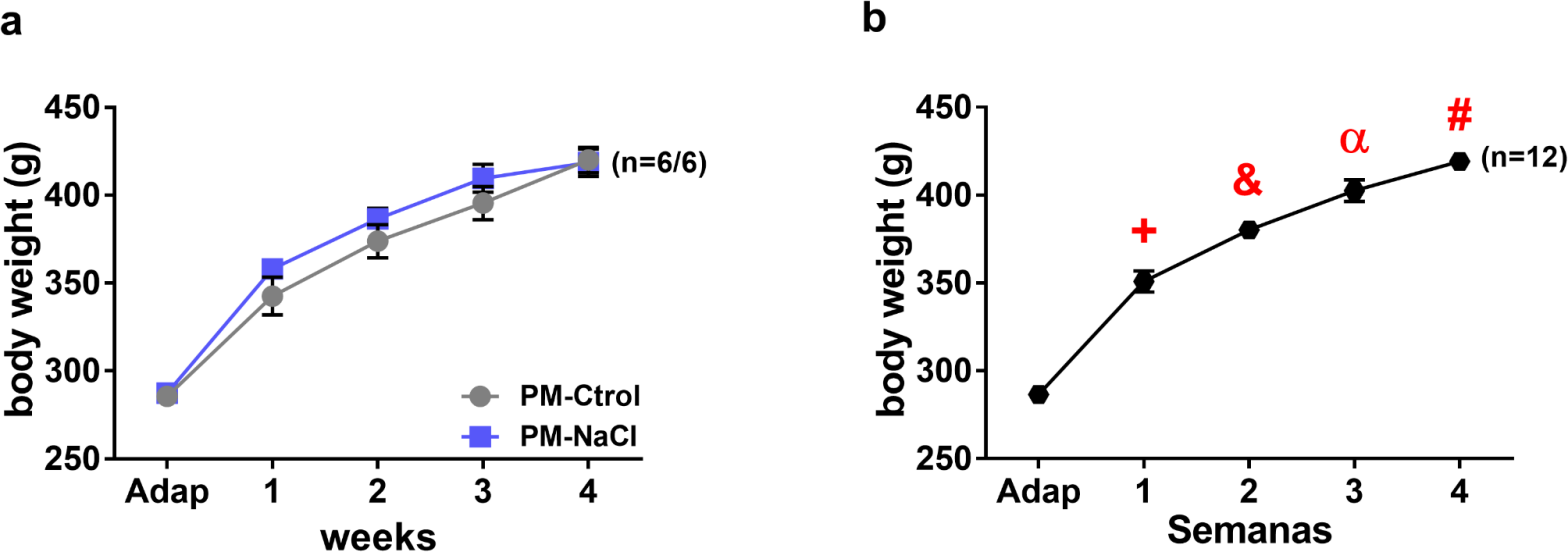
Effects of sodium programming on body size induced by DOCA-salt treatment (25 mg/kg). Average weekly body weight in male PM-Ctrol and PM-NaCl rats (**a**). Average weekly body weight independent of programming factor (**b**). Data express mean ± SE. The numbers in brackets represent the number of animals (*n* = 6 /group). +*p* < 0.05 compared to weeks Adap, 2, 3 and 4. &*p* < 0.05 compared to weeks Adap, 1, 3 and 4. α *p* < 0.05 compared to weeks Adap,1, 2 and 4. #*p* < 0.05 compared to weeks Adap, 1, 2 and 3.

The analysis of the body size (g) of PM-Ctrol and PM-NaCl males during DOCA-salt treatment showed a significant effect of repeated measures (time: F4.40 = 314.63; *p* < 0.001; η_p_^2^ = 0.95) without a perinatal treatment effect (F1.10 = 0.936; *p* < 0.35; η_p_^2^ = 0.085). As can be seen in Fig. 1, the body weight of both groups of animals increased significantly every week throughout the DOCA treatment (Fig. 1a and b).

The brain and heart size did not show a significant change due to DOCA-salt and perinatal treatment [brain (interaction effect: F1.20 = 0.004, *p* = 0.949, η_p_^2^ < 0.001; perinatal effect: F1.20 = 2.867, *p* = 0.106, η_p_^2^ = 0.125; DOCA-salt effect: F1.20 = 0.639, *p* = 0.433, η_p_^2^ = 0.003, Table 1); heart (interaction effect: F1.20 = 1.844, *p* = 0.190, η_p_^2^ = 0.084; perinatal effect: F1.20 = 0.154, *p* = 0.698, η_p_^2^ = 0.007; DOCA-salt effect: F1.20 = 0.715, *p* = 0.408, η_p_^2^ = 0.034, Table 1)].

**Table 1:** Effects of sodium programming on organ size (g) in male PM-Ctrol and PM-NaCl rats after DOCA-salt treatment.

	PM-Ctrol Basal	PM-NaCl Basal	PM-Ctrol DOCA-salt	PM-NaCl DOCA-salt
Brain (g)	0.479 ± 0.011	0.501 ± 0.018	0.470 ± 0.007	0.490 ± 0.010
Heart (g)	0.404 ± 0.025	0.368 ± 0.009	0.393 ± 0.012	0.413 ± 0.029
Kidney (g)	0.414 ± 0.009	0.419 ± 0.009	0.496 ± 0.019*	0.526 ± 0.017*
n	6	6	6	6

Data express mean ± SE. *p < 0.05 significant difference between DOCA-salt groups and basal group.

As can be seen in Table 1, kidney size increased significantly in both groups of animals (PM-NaCl and PM-Ctrol) after DOCA-salt treatment independently of the perinatal effect (DOCA-salt effect: F1.20 = 44.90, *p* < 0.001, η_p_^2^ = 0.692; perinatal effect: F1.20 = 1.62, *p* = 0.217, η_p_^2^ = 0.075; interaction effect: F1.20 = 0.71, *p* = 0.409, ηp^2^ = 0.034).

### Effects of sodium programming on the onset of hypertension induced by DOCA-salt treatment in young adult offspring

**Fig. 2:**
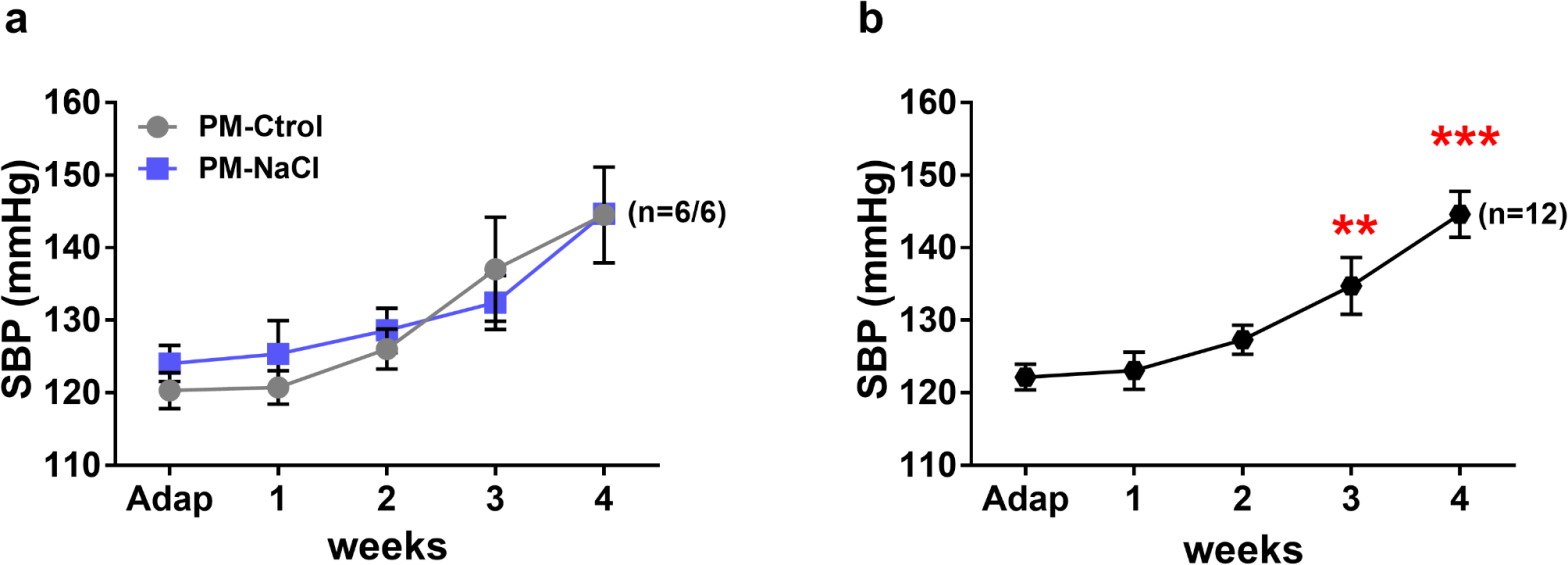
Effects of sodium programming on the onset of hypertension induced by DOCA-salt. (**a**) Average weekly systolic blood pressure (SBP) during DOCA-salt protocol in male PM-Ctrol and PM-NaCl rats. (**b**) Average weekly SBP independent of programming factor. Data express mean ± SE. The numbers in brackets represent the number of animals (*n* = 6/group). ** *p* < 0.05 compared to weeks Adap, 1, 2, and 4. ***p < 0.05 compared to weeks Adap, 1, 2 and 3.

The systolic blood pressure (SBP) analysis in male PM-Ctrol and PM-NaCl rats showed a significant effect of repeated measures: time (F 4.40 = 17.265; *p* < 0.001; η_p_^2^ = 0.63, Fig. 2), but there was no significant effect of the programming factor. As shown in Fig. 2a, DOCA-salt treatment induced an increase in SBP every week, reaching hypertension value in both groups of rats. In turn, the pressure in week 3 of treatment with DOCA was significantly higher than in previous weeks (adaptation, week 1and 2), and in week 4 of DOCA-salt treatment it was significantly higher than in week 3 (Fig. 2b).

### Effects of sodium programming on the pattern of sodium intake induced by DOCA-salt treatment in young adult offspring

The analysis of 1% NaCl intake in male PM-Ctrol DOCA-salt and PM-NaCl DOCA-salt rats showed a significant effect of the perinatal treatment factor (F1.10 = 11.907; *p* = 0.006; η_p_^2^ = 0.54) and of the repeated measures: time (F3.30 = 9.539; *p* < 0.001; η_p_^2^ = 0.48), without interaction between the factors.

**Fig. 3:**
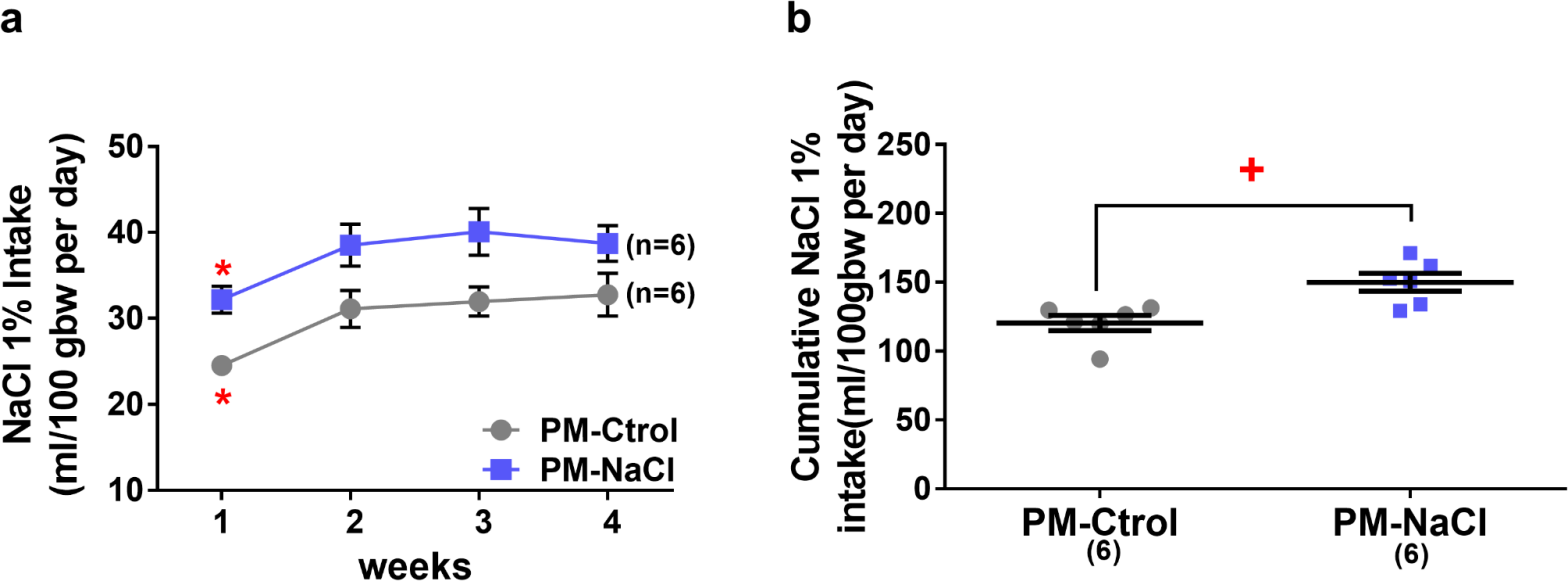
Effects of sodium programming on sodium intake induced by DOCA-salt. Average weekly sodium intake during DOCA-salt protocol in PM-Ctrol and PM-NaCl groups (**a**). Cumulative sodium intake independent of treatment weeks (**b**). Data express mean ± SE. The numbers in brackets represent the number of animals (*n* = 6/group). *p < 0.05 week 1 compared to weeks 2, 3 and 4. +*p* < 0.05 PM-Ctrol compared to PM-NaCl groups.

As can be seen in Fig. 3a and b, sodium intake increased significantly in both groups from week 2 onwards and remained high for the rest of the time. However, animals with maternal programming showed higher sodium intake than PM-Ctrol regardless of treatment time (37.49 ± 1.52 vs. 30.09 ± 1.52) (Fig. 3b).

### Effects of sodium programming on brain *Agtr1a* and *Htr2c* mRNA expression along subfornical organ (SFO) and paraventricular nucleus (PVN) induced by DOCA-Salt treatment in young adult offspring

**Fig. 4:**
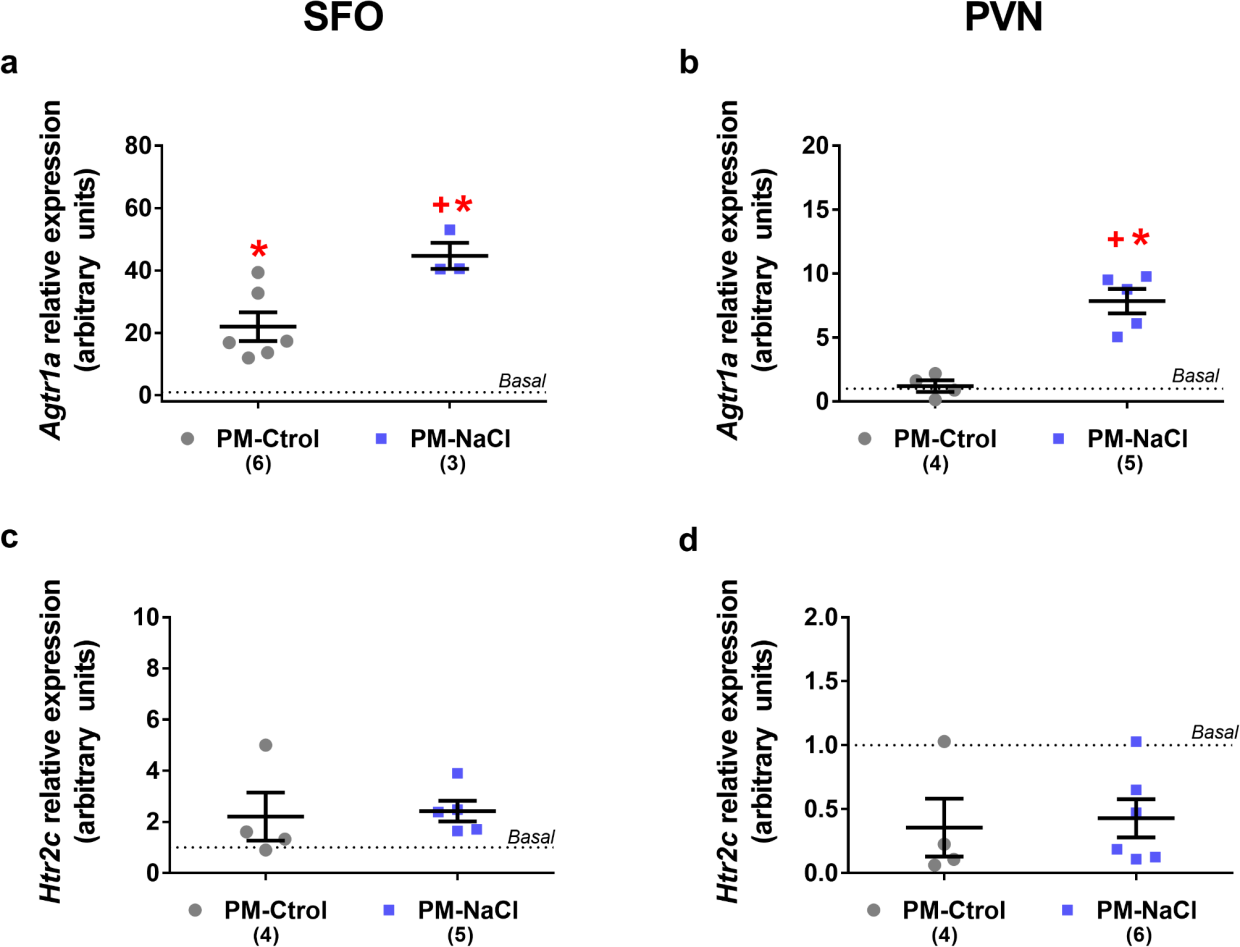
Effects of sodium programming on brain mRNA expression of receptor potential angiotensin receptor (*Agtr1a*) and serotonin 2c receptor (*Htr2c*) in response to DOCA-salt. Relative mRNA levels of *Agtr1a* (**a**-** b**) and *Htr2c* (**c**-** d**) in SFO (**a**,** c**) and PVN (b, d) of male PM-Ctrol and PM-NaCl animals under DOCA-salt conditions. Data express mean ± SE. The numbers in brackets represent the number of animals (*n* = 3–6 /group). The dotted line indicates the mean of basal groups run simultaneously with the respective experimental groups. *p < 0.05 compared to basal group. +*p* < 0.05 PM-NaCl compared to PM-Ctrol groups.

Throughout the SFO, we found a significant increase in *Agtr1a* mRNA expression induced by DOCA-salt treatment. At the same time, *Agtr1a* mRNA expression in PM-NaCl DOCA-salt animals showed higher expression than in the PM-Ctrol DOCA-salt group (F2.10 = 22.23; *p* < 0.001; η_p_^2^ = 0.82). *Htr2c* gene expression in the SFO did not show any significant effects (F2.10 = 1.046; *p* = 0.386; η_p_^2^ = 0.17) (Fig. 4a and c).

Along the PVN, we observed that *Agtr1a* mRNA expression in PM-NaCl DOCA-salt animals was significantly higher than in the PM-Ctrol DOCA-salt group, and in basal animals (without DOCA-salt treatment, baseline) (F2.11 = 7.83; *p* = 0.007; η_p_^2^ = 0.59). *Htr2c* mRNA expression in PVN showed no significant effects (F2.12 = 0.63; *p* = 0.54; η_p_^2^ = 0.09) (Fig. 4b and d).

### Effects of sodium programming on renal *Agtr1a*, *Agtr2*, *Trpv1*, *Avpr2*, and *ENaC* mRNA expression induced by DOCA-Salt treatment in young adult offspring

**Fig. 5:**
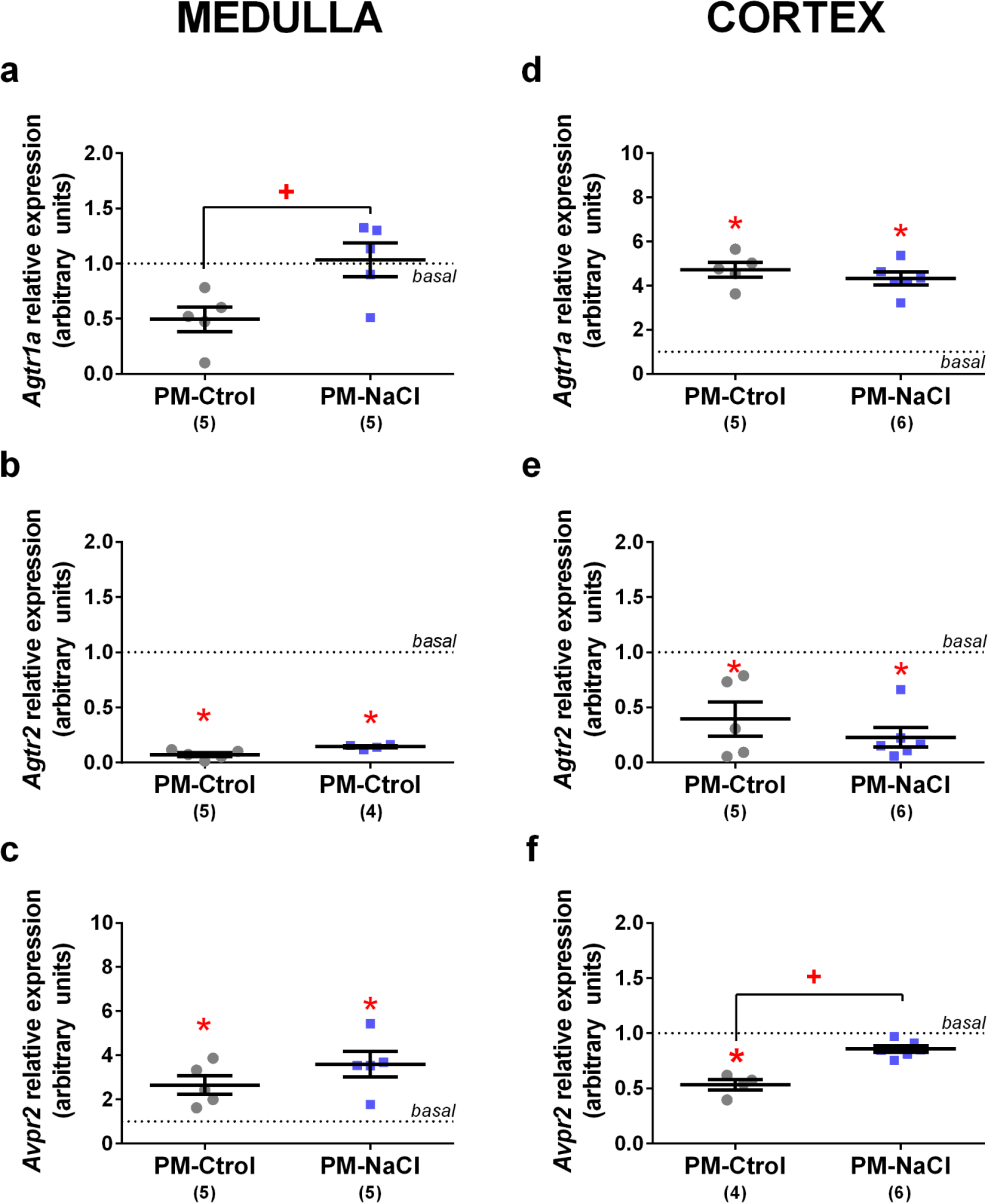
Effects of sodium programming on renal mRNA expression of angiotensin receptor type 1a (*Agtr1a*), angiotensin receptor type 2 (*Agtr2*), vasopressin receptor type 2 (*Avpr2*), in response to DOCA-salt. Relative mRNA levels of *Agtr1a* (**a**,** d**), *Agtr2*(**b**,** e**) and *Avpr2* (**c**,** f**) in renal medulla (**a**-**c**) and cortex (**d**-**f**) of male PM-Ctrol and PM-NaCl animals under DOCA-salt conditions. Data express mean ± SE. The numbers in brackets represent the number of animals (*n* = 4–6/group). The dotted line indicates the mean of basal groups run simultaneously with the respective experimental groups. +*p* < 0.05 PM-NaCl compared to PM-Ctrol. * *p* < 0.05 compared to basal group.

**Fig. 6:**
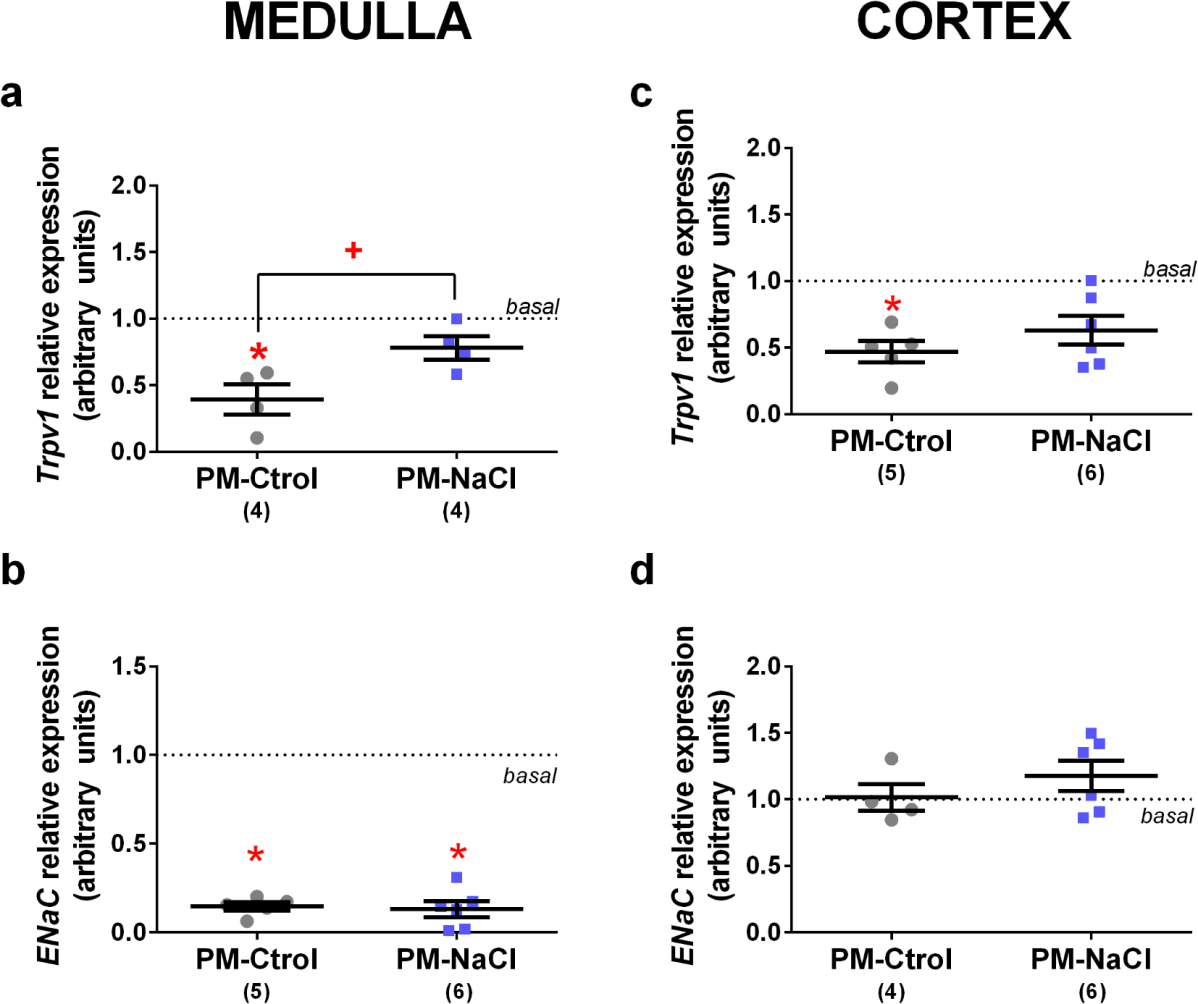
Effects of sodium programming on renal mRNA expression of transient receptor potential vanilloid 1 (*Trpv1*) channel and epithelial sodium channel (ENaC) in response to DOCA-salt. Relative mRNA levels of *Trpv1*(**a**,** c**) and *ENaC* (**b**,** d**) in renal medulla (**a**-**b**) and cortex (c-d) of male PM-Ctrol and PM-NaCl animals under DOCA-salt conditions. Data express mean ± SE. The numbers in brackets represent the number of animals (*n* = 4–6/group). The dotted line indicates the mean of basal groups run simultaneously with the respective experimental groups. +*p* < 0.05 PM-NaCl compared to PM-Ctrol. * *p* < 0.05 compared to basal group.

- *Agtr1*a: In the renal medulla, there was a significant effect between groups (F2.10 = 5.856, *p* = 0.021, η_p_^2^ = 0.54). As can be Fig.  5a, *Agtr1a* mRNA expression in PM-Ctrol DOCA-salt animals was lower than in the PM-NaCl DOCA-salt group. In the renal cortex, *Agtr1a* gene expression in DOCA-treated animals was significantly higher than basal level (F2.11 = 34.473, *p* < 0.001, η_p_^2^= 0.86. Fig. . 5d), regardless of perinatal programming manipulation.

- *Agtr2*: In both the renal medulla and the cortex, *Agtr2* expression in DOCA-treated animals was significantly lower than basal level (medulla: F2.9 = 592.32, *p* < 0.001, η_p_^2^=0.99, Fig.. 5b; cortex: F2.11 = 10.070, *p* = 0.003, ηp2 F= 0.66. Fig. . 5e), without perinatal programming effect.
- *Avpr2*: Along the renal medulla, *Avpr2* mRNA expression in DOCA-salt treated animals was significantly higher than basal level (F2.10 = 5.564, *p* = 0.024, η_p_^2^= 0.53. Fig. 5c). In the cortex, a significant difference in *Avpr2* mRNA expression was observed between groups (F2.10 = 327.889, *p* < 0.001, η_p_^2^ = 0.85). As seen Fig. 5f, the PM-Ctrol DOCA-salt group showed lower mRNA expression than PM-NaCl DOCA-salt animals and basal level.
- *Trpv1*: In the renal medulla, there was a significant difference between the groups (F2.9 = 10.273, *p* = 0.006, η_p_^2^=0.72. Fig. 6a). *Trpv1* expression in PM-Ctrol DOCA-salt animals was significantly lower than in PM-NaCl DOCA-salt and basal level animals. At cortex level, the *Trpv1* channel mRNA expression showed a significant effect between the groups (F2.11 = 6.357, *p* = 0.015, η_p_^2^=064. Fig. 6c), *Trpv1* expression in PM-Ctrol DOCA-salt animals was significantly lower than basal level.

- *ENaC*: In the renal medulla, *ENaC* expression showed a significant effect of DOCA treatment (F2.11 = 141.320, *p* < 0.001, ηp2 =0.96. Fig. 6b), regardless of perinatal programming manipulation. The channel expression in DOCA-treated groups was significantly lower than basal level. At cortex level, in *ENaC m*RNA expression, the ANOVA showed no significant effects (F2.10 = 0.677, *p* = 0.530, ηp2 =0.12, Fig. 6d).

## Discussion

This study examined the vulnerability to the development of hypertension of animals early exposed to voluntary consumption of hypertonic sodium during the perinatal stage, using a neurogenic model of hypertension, deoxycorticosterone acetate, and high-salt diet (DOCA-salt). While the present results shed no light on differential responses in the development of hypertension in animals early exposed to a hyper-sodium environment, the model has allowed us to characterize other aspects of the phenotype induced by perinatal programming, such as salt consumption and changes in gene expression in the brain (AT1R) and kidney (AT1R, V2R and TRPV1) under a hypertensive challenge.

DOCA administration involves direct actions consistent with high levels of aldosterone on mineralocorticoid receptors (MR)^20,21^ binding to MRs expressed in a variety of tissues including the brain, distal convoluted tubule, connecting tubule, and collecting duct of the kidney, to induce thirst, sodium appetite, salt and water retention^22^. These, coupled with the ingestion of 1% NaCl solution as the only beverage, induce a sustained increase in BP, generating hypertension. It has been observed in other perinatal models in which the animals are exposed early to a fat- and sugar-rich diet that does not modify the birth weight of the offspring (just like our perinatal programming model), that DOCA-salt treatment produces an exacerbated increase in BP relative to the control or standard diet group, suggesting that perinatal programming has a facilitating effect on the development of hypertension^23^. However, in our present study, all adult offspring (PM-NaCl and PM-Ctrol) increased BP during the weeks of treatment and became hypertensive, but no effects were evident relative to perinatal programming. This may be due to factors such as age and/or dose of DOCA treatment.

Regarding the age at which the hypertensive challenge was performed, in the case of our work, treatment was initiated in very young animals, at 50 postnatal days (PD). During that stage, rats have high performance in the homeostatic systems, compensating for and possibly masking the differential control of blood pressure induced by the perinatal treatment (as we previously observed after an acute stimulus such as sodium overload)^16^. In most studies, DOCA treatment initiates after 10 PD weeks or more^23–25^. In addition, the spontaneous increase in blood pressure in animals programmed by perinatal forced high sodium intake appears, in general, after 5 months of age^26^. On the other hand, regarding the dose of DOCA threshold, the Mukerjee et al.,^23^ study observed early programming effects on hypertension, using a dose of DOCA lower than the established dose for hypertensive treatment (0.75 mg/kg vs. 1 mg/kg). In our study, the differences may not have appeared due to the supra-threshold dose of DOCA in both groups.

The evolution of the body weight of the animals after DOCA treatment showed the same pattern as the blood pressure, where we observed an increase in weight over the weeks, regardless of the perinatal treatment. In addition, we observed an increased kidney weight after DOCA-salt treatment, as previously described^27–30^, possibly due to the glomerulosclerosis induced by inflammatory factors.

The RAS along the circumventricular organs and hypothalamus is a key part of the regulatory pathway, possibly contributing to the hypertensive phenotype induced by DOCA-salt treatment. In the present study, we observed that DOCA-salt treatment increased salt appetite in both experimental groups (PM-NaCl and PM-Ctrol). This matches multiple reports in the literature that mineralocorticoids per se, such as aldosterone hormone or DOCA administration, induce salt appetite^41^, in both adrenalectomized and intact animals^31,32^. However, in our experiment, sodium intake was significantly higher in animals with perinatal programming irrespective of the time of DOCA administration. This result follows our previous data where, in PM-NaCl animals, sodium deficiency induces a greater preference for sodium and consequent consumption of a more hypertonic cocktail than in control animals. These animals also showed decreased activity along the nucleus of the solitary tract (NTS)^33^, associated with the inhibition of sodium appetite^34,35^. On the other hand, the central regulation of sodium consumption mainly involved the excitatory effect of ANGII on sodium appetite by its binding to the AT1R receptor^17,18,36^, and the central serotonin system that inhibits this behavior through its receptor, 5HT2CR^18,19,34,37^. The receptors of both antagonistic systems colocalize in critical structures such as the SFO and hypothalamic PVN to modulate sodium intake and volemia^18^. Therefore, the observed increased of *Agtr1a* expression in these regions involved after DOCA-salt treatment may influence the increased sodium consumption observed in sodium-programmed animals. In agreement with our findings, the influence of early environment on brain RAS has also been demonstrated, for example, by Mukerjee et al.,^23^, who observed an increase in hypothalamic AT1R mRNA after DOCA treatment in adult offspring perinatally exposed to a high fat and sugar diet. Also, hypertension induced by a prenatal low-protein diet increases AT1R mRNA expression along the SFO^38^. These data together suggest that altered salt intake by sodium-programmed animals may be due, at least in part, to brain angiotensin mechanisms (AT1R) and activity changes in the brain control circuitry of sodium appetite.

Intrarenal RAS is viewed as another piece of the pathology of hypertension, regardless of the systemic RAS, as it may induce sodium retention and inflammatory effects^39^. The angiotensin receptors, AT1R and AT2R, are expressed in the renal medulla and cortex, with AT1R being more abundant than AT2R. The function of both receptors is antagonistic; in the cortex area they mainly modulate glomerular filtration and in the medulla they regulate sodium reabsorption mechanisms. Our data showed, in the kidney cortex, an upregulation of the mRNA of AT1R receptor in DOCA-treated rats, as previously described by Oishi et al.,^28^ and other kinds of perinatally programmed hypertension (such as a low protein diet)^40,41^, presumably reflecting the upregulated state of the receptors in the model, in which renin and ANG II are suppressed. However, in the renal medulla, early sodium exposure produced a refractory response to DOCA in AT1R mRNA expression in adult offspring compared to control animals (which decreased its expression in relation to the basal level, Fig. 5), showing expression close to the basal level, possibly affecting urine concentration at this level. On the other hand, the natriuretic effect of AT2R activation is mediated by the signaling of downstream messengers that internalize and inactivate the main Na + transporter molecules, Na+-H+-exchanger-3 (NHE-3) and Na+-K+-ATPase (NKA), counteracting the actions of ANGII-AT1R, to increase Na + reabsorption by stimulating these transporters mainly in the proximal tubule^42^. Consistent with this, we observed a downregulation of AT2R throughout the renal cortex after DOCA treatment, opposite to the AT1R expression. However, unlike AT1R expression in the medulla, AT2R expression was unaffected by perinatal programming, altering AT1R/AT2R balance in the PM-NaCl animals in this region.

Hypertension induced by DOCA-salt treatment involves the plasma enhancement of vasopressin to promote water reabsorption and vascular constriction^43–46^. The present study also demonstrated upregulation of the V2 receptor along the renal medulla, where it has a key role in hydroelectrolyte control, which possibly allows water reabsorption through its control of aquaporin 2, modulating the hypervolemia produced by the DOCA-salt hypertensive model^45,46^. On the other hand, a decrease in the expression of the V2 receptor is observed in the renal cortex in PM-Ctrol animals; this downregulation of V2 receptors in DOCA-salt-treated hypertensive animals was previously reported by other authors and was associated with the increase in plasma AVP concentration^44,46,47^. However, early sodium exposure to hypertonic saline did not produce any response to DOCA compared to PM-Ctrol along the renal cortex. These results agree with our previous findings that the central vasopressinergic system is also affected by perinatal exposure, which slows the increase in the expression of the neuropeptide AVP and the expression of total and synthesized de novo (heteronuclear) RNA of AVP after the infusion of hypertonic NaCl solution^16,48^ The side effect of this refractory response to AVP may be a reduced ability to recover renal water to restore osmolarity, altering normal function and/or the mechanisms involved in achieving fluid and electrolyte homeostasis.

Our data also showed in PM-Ctrol a downregulation of expression of channel involved in the anti-inflammatory effect induced by DOCA treatment, such as Trpv1 in the renal cortex. However, sodium programmed animals did not show this change in Trpv1 expression possibly altering the hypertensive pattern induced by DOCA-salt treatment. In the same way, at the renal medulla, the *Trpv1* expression decreased significantly after DOCA treatment in PM-Ctrol animals in comparison with PM-NaCl and basal, indicating a differential programming effect in this channel. According to previous data, the renal TRPV1 channel has been considered a protective buffer to the blood pressure response after a sodium overload challenge, as its activation decreases the blood pressure and renal injuries induced by the DOCA-salt hypertension model, diminishing inflammatory responses^13,49^. Trpv1 KO mice presented a more aggravated inflammatory response to DOCA-salt treatment than wild type^25^. The present results regarding the renal expression of *Trpv1* showed that programmed animals are refractory to the phenotype induced by DOCA treatment.

Sodium reabsorption is one of the main mechanisms in pressure-natriuresis regulation and is carried out by different sodium channels. Manning et al., 2002^50^ reported a significant increase of ≈ 150–300% in two apical sodium cotransporters (NA-K-2Cl and NA-K) in prenatally programmed hypertension but did not observe any difference in the ENaC subunits, which mediated sodium reabsorption in the collecting duct. Similarly, we found in the present study a reduced expression of ENaC induced by DOCA-salt treatment along the renal medulla but without a perinatal programming effect, suggesting that the high sodium environment resulting from the DOCA protocol may produce a negative feedback effect on ENaC expression to avoid excess volume expansion, as was recently reported by Pitzer et al., 2020^51^.

Among the mechanisms by which early life events influence the development of hypertension, it seems that the number of glomeruli, and molecular changes in post-glomerular segments of the nephron that participate in sodium handling, play an important role^6^. Our recent study showed that the natriophilia proper to the perinatal period reduced the number of glomeruli and affected blood pressure regulation after an osmotic challenge^16^, but also differentially affected the expression of molecules involved in the renal sodium balance, such as AT1R and TRPV1. However, under this acute challenge, the urinary response of sodium excretion was the same in sodium-programmed animals as in the control group^48^. The present data add evidence about the renal changes in the expression of different molecules that modulate the hypertensive phenotype induced by DOCA-salt protocol (chronic challenge), showing less response in AT1R, V2R and TRPV1 expression in PM-NaCl animals during pharmacological stimulus, similar to the basal condition. These changes possibly affect sodium kidney management during the salt overload of the DOCA-salt protocol, which, together with the exacerbated sodium intake of these animals, could affect the cumulative sodium intake, resulting in body sodium imbalance as observed in other perinatal programming models^52^. However, these changes also could compensate, by means of increased sodium excretion, the increased sodium intake observed in PM-NaCl animals during DOCA treatment, avoiding a higher hypertensive response as an adaptative mechanism of early exposure to a rich sodium environment, as reported in other models^53^. Unfortunately, this is merely speculative, as urine response could not be determined in the present study. Thus, renal physiological performance opens a new perspective for study in the field of programming voluntary perinatal sodium consumption.

In sum, the effects of perinatal imprinting induced by voluntary hypertonic sodium intake produced a marked increase of salt intake in adult offspring after DOCA treatment. This increase was accompanied by increases in AT1R receptor expression at the brain level in key areas of the modulation of salt appetite and autonomic function, such as the SFO and the hypothalamic PVN, and, at the renal level, by an alteration of reabsorption-inflammation-promoting mechanisms relative to excretion-anti-inflammation-promoting mechanisms, with programmed animals being less responsive to AT1R, Trpv1, and V2R gene expression induced by DOCA drug treatment, in the medulla or cortex respectively. These changes possibly made the organisms more susceptible to the development of chronic non-communicable diseases, such as hypertension and other conditions mainly caused by changes in sodium intake and/or the regulatory mechanisms of the angiotensin and vasopressin systems.

## Methods

### Animals

Female and male adult Wistar-derived rats, born and reared in the breeding colony of the Instituto Ferreyra (INIMEC-CONICET, UNC, Córdoba, Argentina) were used in these experiments. Females weighing 230–260 g and males 350–380 g, 60 days old and not littermates, were individually housed in standard holding chambers (40 × 40 × 70 cm).

**Fig. 7:**
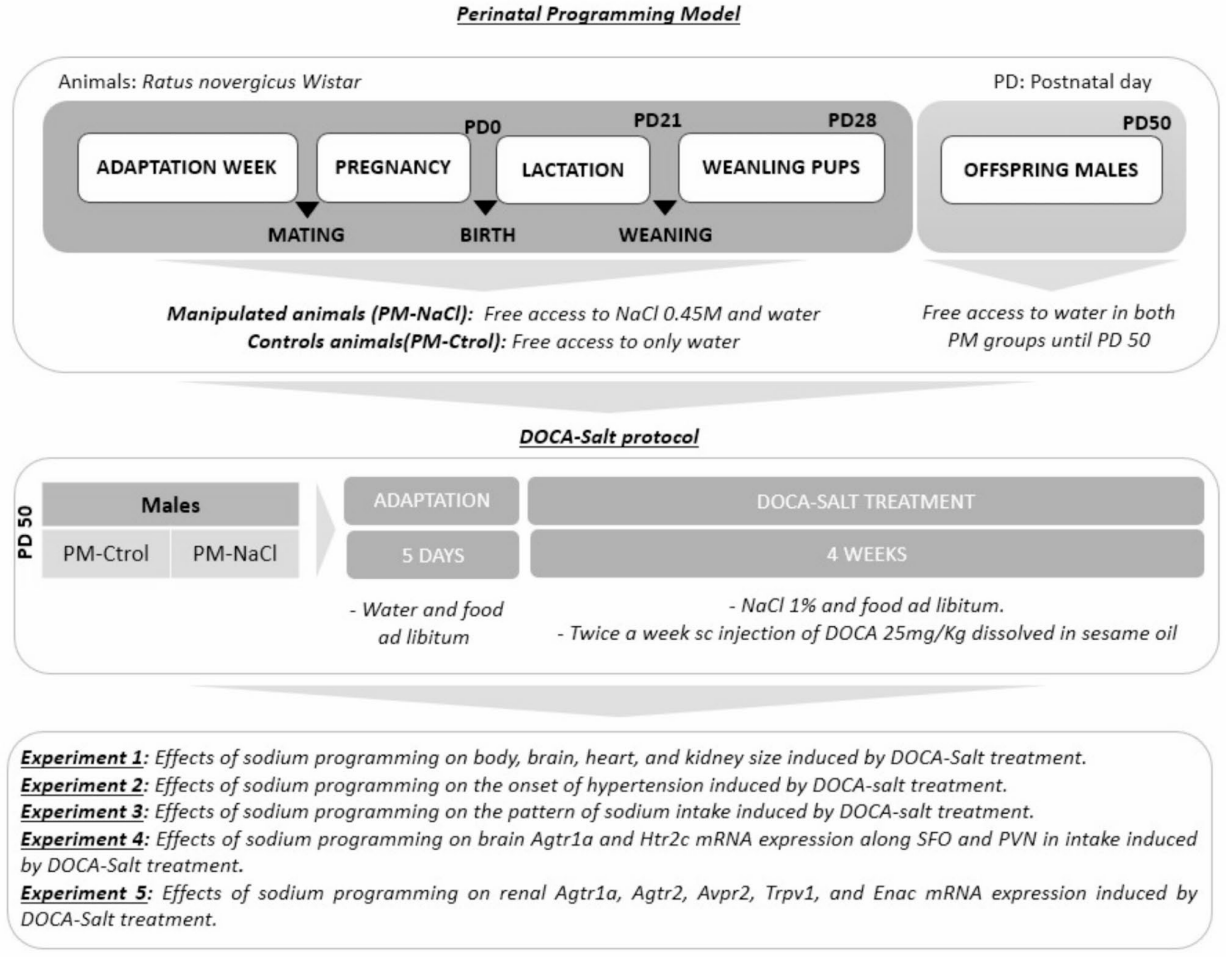
Schematic diagram showing the conditions of the dams and their offspring from adaptation week until the beginning of the studies. From postnatal day (PD) 28 until PD50, male offspring of PM-NaCl and PM-Ctrol groups were kept in standard conditions. On PD50 and after 5 days adaptation, DOCA-salt hypertension was induced by two subcutaneous weekly injections of 25 mg/kg of deoxycorticosterone acetate (DOCA) and 1% NaCl to drink instead of tap water for four weeks. Experiment 1, we analyzed the effects of sodium programming on body, brain, heart, and kidney size (g) induced by DOCA-salt treatment. Experiment 2, the systolic blood pressure (SBP) was recorded twice every week during DOCA-salt treatment in conscious rats using the non-invasive tail-cuff method. Experiment 3, we analyzed the effects of sodium programming on sodium intake induced by DOCA-salt treatment. Experiments 4 and 5, the brains (experiment 4) and kidneys (experiment 5) were collected for determination of gene expression induced by DOCA-salt.

Room lights were on for 12 h/day from 08:00 a.m., and temperature was controlled at 23 ºC ± 1. All experimental protocols were approved by INIMEC’s appropriate animal care and use committee (Res #009/2019), following the guidelines of the international Public Health Service Guide for the Care and Use of Laboratory Animals (NIH Publications No. 8023, revised 1978). We complied with the ARRIVE guidelines. The protocol was executed according to Porcari et al., 2023 and Macchione et al., 2012, 2015. Briefly, 7 days before mating, female rats were randomly divided in two groups: one group without perinatal manipulation (PM) (Female/Male PM-Ctrol group) with free access to distilled water and normal sodium diet (Purina rat chow, Argentina, containing approx. 0.18% NaCl), and the other group (Female/Male PM-NaCl group) which, in addition to free access to distilled water and normal sodium diet, had voluntary access to a hypertonic NaCl solution (0.45 M NaCl) (Fig. 7). After one week of adaptation, one couple per cage was placed for mating in the same standard holding chamber until found sperm-positive, maintaining hypertonic NaCl solution access in the PM-NaCl group. When pregnancy was confirmed (1–5 days), males were removed. Pregnant rats were maintained in the same holding chamber. Within 24 h after birth, litters were culled to ten pups, retaining both males and females in each litter. Litters with fewer than six pups were not included. Dams continued to receive their respective manipulation conditions until pups were weaned at postnatal day 21–22 (PD 21–22). After weaning, male pups were separated from female pups, and were maintained in the same conditions as their dam until reaching PD 28. Then, pups of both experimental conditions were kept in standard conditions of water and food until about 50 days of age (PD 50), when they were assigned to the corresponding experiments (see experimental protocols). The experiments were performed only in males which previously demonstrated alteration in blood pressure regulation^16^.

### DOCA-salt hypertensive rats

DOCA-salt hypertension was induced by two subcutaneous weekly injections of 25 mg/kg of deoxycorticosterone acetate (DOCA) dissolved in sesame seed oil and 1% NaCl to drink instead of distilled water for four weeks. Body weight and NaCl consumption were determined twice a week for all rats (six Males PM-Ctrol and six males PM-NaCl). They were not uninephrectomized^54,55^.

### Measurement of systolic blood pressure

The systolic blood pressure (SBP) was recorded twice every week during treatment in conscious rats using a non-invasive tail-cuff method (AD Instrument Power Lab Data Acquisition System, Australia). Animals were trained with instruments for one week before blood pressure detection. They were placed in a heated restrainer at 37 ± 1 °C for 10 min during measurements.

Rats were restrained and allowed to rest for 10 min before the tail-cuff was inflated to 250 mmHg and slowly deflated over a period of 15 s. For each rat, 4 blood pressures were measured and their average was taken as the SBP. At the end of this experiment, rats were euthanized by decapitation for mRNA analysis.

### Relative mRNA expression in brain areas and Kidney

Immediately after decapitation, male offspring brains and kidneys were weighed, collected, and frozen on dry ice in RNAse-free conditions, and stored at -80 ºC for Gapdh, *Htr2c*, *Agtr1a*, *Agtr2*, *Trpv1*, A*vpr2*, and *ENaC* RNA determinations by qPCR assay.

We performed transversal sections of 1500 μm for kidney cortex (mainly involved in filtration procedure) and medulla (mainly involved in concentrate/dilute urine). Despite the regional separation performed, there is an anatomic-functional subdivision within the renal cortex and medulla which, for technical reasons, it was not possible to address. Therefore, the cortex section analyzed in the present study included a region in which proximal tubules predominate together with another mainly composed of distal tubules. The medulla region also included two functionally different regions: the external medulla and the internal medulla, in which sodium transport mechanisms and their regulation differ.

For the brain, we performed coronal sections of 1320 μm for the subfornical organ (SFO; bregma: -0.8 to -1.4 mm) and the paraventricular nucleus (PVN, bregma: -1.44 mm to -1.92 mm). These were obtained from the frozen kidneys and brains through microtome cuts, and punches were performed using a stainless-steel needle (inner diameter 1.5 mm). The brain nuclei were identified and delimited according to a rat brain atlas^56^.

RNA was isolated from the punched kidney and brain tissue using Trizol reagent (Invitrogen, Carlsbad, CA, USA), as directed by the manufacturer with some modifications: RNA precipitation with isopropanol was performed overnight at − 20 °C. Dnase-treated (Fermentas) RNA was quantified using a NanoDrop 2000 UV-Vis spectrophotometer, and was then reverse-transcribed into cDNA (enzyme RTM-MLV – Promega). The gene expression was analyzed to *Gapdh*, *Agtr1a*, *Agtr2*,* Trpv1*, *Avpr2* and *ENaC* in the kidney, and *Gapdh*, *Agtr1a* and *Htr2c* in the brain. The expression was determined using Syber Green Real-Time PCR Master Mixes (Applied Biosystems™) in the Step One Real-Time equipment (StepOne™ Real-Time PCR System #4369074, Applied Biosystems, Foster, CA, USA). Primer sequences are shown in Table 2.

**Table 2:** Primer pairs for *Gapdh*,* Agtr1a*,* Agtr2*,* Avpr2*,* Htr2c*, *Scnn1g* and *Trpv1*.

GENE	GENBANK ACCESS NUMBER	FORWARD PRIMER 5´-3´	REVERSE PRIMER 5´-3´	PRODUCT LENGHT (BP)	ANNEALING TEMP. (°C)	Eff %
***Gapdh***	NM_017008.4	TGTGAACGGATTTGGCCGTA	ATGAAGGGGTCGTTGATGGC	93	60	99
***Agtr1a***	NM_030985.4	AACCCTCTGTTCTACGGC	ACCTGTCACTCCACCTCA	194	56.5	104
***Agtr2***	NM_012494.4	AGAAGGAATCCCTGGCAAGC	GCAATGAGGACAGACAAGCC	70	59.5	99
***Avpr2***	NM_019136.2	AAGCTCCTCTGGAAAGACCC	CAAAGCAGGCTACGCAACTC	127	59.4	103
***Htr2c***	NM_012765.3	TTGGACTGAGGGACGAAAGC	GGATGAAGAATGCCACGAAGG	102	59.6	90
***Scnn1g (ENaC)***	NM_017046.2	CATCAAAGTCCACTTCCAGAAACT	ACAGCACTGTACTTGTAAGGGT	78	59.2	96
***Trpv1***	NM_031982.1	TTCACCGAATGGGCCTATGG	TGACGGTTAGGGGTCTCACT	125	59.9	93

### Calculations of relative gene expression

The relative quantification was determined by the ΔΔCt method, where the fold change of mRNA content in the unknown sample relative to the control group was determined by 2 ^-ΔΔCt^ (ΔΔCt = ΔCt_unknown_ − ΔCt _control_). For each sample, the Ct was determined and normalized to the average of the housekeeping *Gapdh*. This gene is a constitutive and stable gene between groups, which allows its use as a control for this experiment. All samples were run in duplicate with the average Ct used for each sample.

The Ct of the calibrator group (the mean Ct of the naïve male adult rat) was then subtracted from each sample to give a Ct value. Relative quantification of the *Agtr1a*,* Agtr2*,* Avpr2*,* Htr2c*,* Trpv1*, and *Scnn1g* (ENaC gen) gene expression was normalized to the naïve male adult rat. Data are presented as mRNA relative to the control calibrator group.

### Statistical analysis

Results were expressed as mean (M) ± standard error (S.E.) and a level of *p* < 0.05 was considered statistically significant. The normality of the data was assessed with the Shapiro– Wilk test. Blood pressure and heart rate data were analyzed by one-way analyses of variance (ANOVA) with perinatal manipulation (PM-NaCl and PM-Ctrol) as the main factor, and weeks of DOCA treatment (for 4 weeks) as repeated measurements. qPCR results were analyzed by one-way ANOVA, with perinatal manipulation (PM-NaCl and PM-Ctrol) as the main factor.

Analyses were performed using the STATISTICA 8 software. Statistically significant interactions were further analyzed using the Tukey test (type I error probability was set at 0.05). The partial eta squared (ɳ_p_^2^) was used to describe effect sizes of the ANOVAs and was interpreted using the guidelines: small (ɳ_p_^2^ = 0.01–0.05), medium (ɳ_p_^2^ = 0.06–0.13), and large (ɳ_p_^2^ ≥ 0.14))^57^.

## Data Availability

The data presented in this study are available on request from the corresponding author Andrea Godino via email agodino@immf.uncor.edu.

## Abbreviations

5HT2CR: Serotonin 2c receptor
2c: receptor gene
*Agtr1a*: Angiotensin type 1a receptor gene
*Agtr2*: Angiotensin type 2 receptor gene
ANGII: Angiotensin II
ANOVA: One-way analyses of variance
AQP2: Aquaporin-2
AT1R: Angiotensin type 1 receptor
AT2R: Angiotensin type 2 receptor
AVP: Vasopressin
*Avpr2*: Vasopressin V2 receptor gene
BP: Blood pressure
DOCA-salt: Deoxycorticosterone acetate and high-salt diet
ENaC: Epithelial sodium channel
*Gapdh*: Glyceraldehyde-3-phosphate dehydrogenase gene
HTN: Hypertension
*Htr2c*: Serotonin 2c receptor gene
Icv: Intracerebroventricular
MR: Mineralocorticoid receptors
mRNA: Messenger RNA
Na+: Ion sodium
NaCl: Sodium chloride
NA-K: Sodium cotransporter
NA-K-2Cl: Sodium cotransporter
NTS: Nucleus of the Solitary Tract
PD: Postnatal day
PM: Perinatal manipulation
PM-Ctrol: Control group
PM-NaCl: NaCl group
PVN: Paraventricular nucleus of hypothalamus
qPCR: Real time polymerase chain reaction
RAS: Renin-angiotensin system
RNA: Ribonucleic acid
S.E.: Standard error
SBP: Systolic blood pressure
*Scnn1g*: Epithelial sodium channel gene
SFO: Subfornical organ
SIK: Salt inducible kinase
*Trpv1*: Transient receptor potential cation channel gene
TRPV1: Transient receptor potential cation channel
V2R: Vasopressin V2 receptors
